# Stem cells in the treatment of myocardial injury-induced cardiomyopathy: mechanisms and efficient utilization strategies

**DOI:** 10.3389/fphar.2025.1600604

**Published:** 2025-06-18

**Authors:** Jiali Yang, Tian Yue, Jian He, Shiqiang Xiong, Yan Luo, Jun Hou

**Affiliations:** ^1^ Department of Cardiology, The Third People’s Hospital of Chengdu/Affiliated Hospital of Southwest Jiaotong University, Chengdu Institute of Cardiovascular Disease, Chengdu, Sichuan, China; ^2^ School of life science and engineering, Southwest Jiaotong University, Chengdu, Sichuan, China

**Keywords:** stem cells, cardiomyopathy, cell differentiation, paracrine effect, hydrogels

## Abstract

Cardiac tissue injury and repair have always been a research hotspot in the field of cardiovascular disease. Limited and lost myocardial cells are non-renewable, and the current clinical treatment effect is still poor. The stem cells-based treatment strategy for cardiomyopathy is expected to solve the current treatment pain points. A variety of stem cells have the potential to differentiate into cardiomyocytes and form cardiac tissue, and the strong paracrine activity of stem cells also plays an important role in the regulation of inflammation, oxidative stress and cardiomyocyte apoptosis in cardiac tissue. Limited by the survival rate and stem cells activity after stem cells transplantation, the effect of stem cells therapy on cardiomyopathy is still not ideal. Pretreatment of stem cells or genetic modification to enhance the adaptability of stem cells to the environment, or the use of new biomaterials to assist stem cells transplantation is an effective optimization scheme and significantly enhances the therapeutic effect of stem cells therapy for cardiomyopathy. In this review, the types of stem cells widely studied in the treatment of cardiomyopathy, the role of stem cells in the treatment of cardiomyopathy, and how to efficiently use stem cells to treat cardiomyopathy are described in detail, which provides a theoretical basis for promoting the preclinical research and clinical transformation of stem cell therapy for cardiomyopathy.

## 1 Introduction

Cardiomyopathy is a myocardial injury affected by many factors. It involves changes in myocardial structure and function, resulting in abnormal cardiac pumping function, and ultimately the development of heart failure (HF) (Arany, 2024; Ren et al., 2021; Zhang J. et al., 2022). Common ischemic injury caused by acute and chronic myocardial infarction (MI) (Damluji et al., 2021; Dauerman and Ibanez, 2021; Murphy and Goldberg, 2022), ischemia-reperfusion (IR) injury (Ma H. et al., 2022; Xiang et al., 2024), myocardial toxicity from drugs such as adriamycin and alcohol (Song et al., 2021), myocardial injury caused by inflammation or autoimmune diseases, diabetes, or myocardial mechanical injury fromlong-term hypertension (Tan et al., 2020). The pathological process of cardiomyopathy is affected by many factors, including acute and chronic inflammation following injury, resulting in myocardial fibrosis and remodeling. These changes result in decreased myocardial systolic function, thus affecting the heart’s pumping capacity and reducing cardiac output (Hollenberg and Singer, 2021). The treatment of cardiomyopathy primarily involves basic etiological treatment, surgical implantation treatment, pharmacotherapy and lifestyle intervention (Honigberg and Givertz, 2019; Ruberg and Maurer, 2024). However, current treatment plans fail to address the core problems of cardiac structural changes and cardiac function loss in patients (Bogle et al., 2023). With the continuous development of stem cells-based therapies for myocardial injury and heart disease have became as a hot research in recent years (Meng et al., 2018; Ning et al., 2023; Nishiyama et al., 2022). The core of stem cells therapy is to use the regenerative repair potential of stem cells to improve cardiac function and even reverse the course of cardiomyopathy. It has shown great potential for treatment and application in preclinical research on cardiomyopathy (Zhao et al., 2020). Although some studies on cardiac stem cells have yielded disappointing results, other types of stem cells, including induced pluripotent stem cells (iPSCs) (Sayed et al., 2020) and mesenchymal stem cells (MSCs) (Matta et al., 2022), have demonstrated efficacy in treating cardiomyopathy. However, despite the potential shown by stem cells in the treatment of cardiomyopathy myocardial injury, several factors still limit the research and clinical translation. These include cell viability and differentiation capacity, immune rejection, and the need to further explore long-term effects. (Liu and Olson, 2022). Therefore, this review provides a detailed overview of the current stem cells types used for cardiomyopathy therapy, therapeutic mechanisms, limitations, and optimization of stem cells therapeutic regimens, such as genetically modifying stem cells or improving stem cells inhibition protocols, with the aim of providing a detailed theoretical basis for cardiomyopathy stem cells therapy.

## 2 Types of stem cells in the treatment of cardiomyopathy

Research has focused on iPSCs, MSCs, and embryonic stem cells (ESCs). Each type of stem cells possesses distinct characteristics and therapeutic roles that usually need to be determined based on the type of cardiac injury.

### 2.1 iPSCs

iPSCs are stem cells obtained by reprogramming adult cells, including skin-derived or blood-derived cells, with transcription factors to confer pluripotency (Zhang et al., 2021). iPSCs have a high capacity for value-addition and differentiation and can potentially differentiate into a wide range of cell types, including cardiomyocytes, vascular endothelial cells, smooth muscle cells, and bone (Meng and Guo, 2023). A recent study demonstrated that human-derived iPSCs (hiPSCs) can successfully differentiate into cardiomyocytes when exposed to a combination of isulfonic acid and Gremlin 2, a member of the Gremlin family of proteins, exhibiting a high proportion of elongated cells, some of which displayed a remarkable adult AM-like morphology (Tay and Melosh, 2019). And human induced pluripotent stem cells-derived cardiomyocytes (hiPSCs-CM) exhibited similar electrical signaling properties as cardiomyocytes, and upon electrical stimulation, hiPSCs-CM showed distinct atrial morphology action potentials. Also hiPSCs-CM were able to respond to adrenergic stimulation (Ahmad et al., 2023). Xin et al. used highly purified hiPSCs-CMs to treat myocardial infarction (MI) in mice. After a 4-week treatment period, a significant increase in capillary density and a decrease in cardiomyocyte apoptosis were observed. These treatments also led to significant improvements in left ventricular ejection fraction (LVEF), end-diastolic left ventricular internal diameter (LVIDd), and maximum positive and negative pressure derivatives (±dp/dt) in MI mice (Jiang et al., 2020; Wu et al., 2024).

As the source of iPSCs is identified, they can usually be obtained from reprogramming of the patient’s own cells, avoiding the risk of short-term or long-term rejection, and ensuring the safety needs of iPSCs in research and promotion. Tobias Deuse et al. obtained mouse engineered iPSCs through lentiviral transfection, and during a 5-day transplantation of homozygous mice, there was no significant increase in mouse interferon γ (IFN-γ) as well as interleukin (IL)-4 suggesting that immunosuppression did not occur. In contrast, homozygous mice showed a strong increase in IFN-γ and IL-4 expression, and only homozygous mice showed a robust IgM antibody response against mouse iPSCs (homozygous mice did not) (Deuse et al., 2019).

The excellent differentiation ability has enabled IPSCs to show some pro-tissue repair ability in cardiomyopathy disease treatment and has also provided a research basis for establishing *in vitro* cardiac disease models, especially for some cardiac diseases caused by genetic defects. In the study of dilated cardiomyopathy (DCM) with mutations in RNA-binding motif protein 20 (RBM20), Takahiko Nishiyama et al. constructed a genetically defective mouse model of DCM using adenine base editing (ABE) and primer editing (PE), which provided an experimental basis for the study of DCM (Nishiyama et al., 2022).

iPSCs show great potential in heart disease research and therapy. They can be generated through the reprogramming of adult cells using transcription factors and possess excellent differentiation potential, allowing them to generate a wide range of cell types such as cardiomyocytes, vascular endothelial cells, and smooth muscle cells. iPSCs-derived cardiomyocytes are like adult cardiomyocytes in electrophysiological properties and are able to exhibit normal cardiac function in response to electrical and hormonal stimulation, which provides a new direction for cardiac repair. The source of iPSCs is usually from the patient’s own cells, which effectively avoids the risk of immune rejection and enhances the safety of their clinical application. iPSCs are also important in constructing models of genetic defects, especially in the study of cardiac diseases caused by genetic mutations, such as DCM.

### 2.2 MSCs

MSCs are derived from bones, adipose tissue, umbilical cord, or dental pulp, etc.,(Bian et al., 2022; Ma T. et al., 2022; Yan et al., 2023). Depending on their origin, MSCs differ in their ability to differentiate, and usually have the potential to differentiate into bone, cartilage, or adipocytes. However, current studies have shown that MSCs usually do not have the ability to differentiate directly into cardiomyocytes, and therefore, current studies on the use of MSCs for cardiomyopathy therapy have focused on the paracrine effects of MSCs in the therapeutic effects of cardiomyopathy (Wu et al., 2023). Numerous preclinical and clinical studies have shown that MSCs release large amounts of anti-inflammatory, anti-fibrotic, and growth factors that protect cardiomyocytes from apoptosis. Additionally, MSCs secrete a variety of non-coding RNAs after transplantation. These mechanisms enable MSCs transplantation to play an important role in the prevention and treatment of cardiomyopathy, including MI, ischemic heart failure, myocardial inflammation, and drug-induced myocardial injury (Zhang Z. et al., 2022). In a recent study by Xue Wang et al., adipose-derived MSCs and their secreted exosomes were found to significantly increase cardiomyocyte viability and reduce cardiomyocyte apoptosis in a MI model by directly targeting transforming growth factor beta receptor 2 (TGF-βR2) and reducing the phosphorylation of Smad protein family member 2 (Smad2), as well as exhibiting extremely strong *in vivo* and *in vitro* anti-inflammatory and anti-fibrotic capacity *in vivo* and *in vitro* (Wang et al., 2021). In another study, Ronald J Vagnozzi et al. found that direct intracardiac injection of MSCs induced CCR2^+^ and CX3CR1^+^ macrophage accumulation and successfully partially restored cardiac viability in ischemia-reperfusion-injured hearts, by selectively inducing macrophage aggregation, MSCs ameliorated local inflammation and improved the activity of cardiac fibroblasts, reduced extracellular matrix (ECM) collagen deposition, and improved fibrosis while enhancing the mechanical properties of the injured part. It was suggested that the therapeutic effect of MSCs was realized based on the pathway related to wound healing in the acute inflammatory phase (Vagnozzi et al., 2020). Thus, although MSCs have a limited capacity for direct cardiomyocyte differentiation, their potent secretory capacity also provides a therapeutic effect for cardiomyopathy.

Although MSCs have limitations in direct differentiation into cardiomyocytes, their therapeutic potential through paracrine effects is particularly prominent in cardiomyopathy therapy. MSCs were able to significantly improve cardiomyocyte survival, reduce apoptosis, and promote cardiac tissue repair by secreting a variety of anti-inflammatory and anti-fibrotic factors as well as non-coding RNAs. Transplantation of MSCs can restore cardiac function by ameliorating the local inflammatory response and fibrotic process in the treatment of a variety of cardiac diseases, including ischemic heart disease, myocarditis, and myocardial infarction. MSCs also play an important role in wound healing during the acute inflammatory phase by inducing macrophage accumulation and selectively modulating the immune response.

### 2.3 ESCs

ESCs are derived from early embryos and have an extremely high value-added capacity and many functionalities (Wang et al., 2022). Numerous studies have shown that ESCs have a strong ability to differentiate into cardiomyocytes and can differentiate into various types of cardiac cells and provide strong cellular support for repair of damaged myocardium (Chen et al., 2020; Guénantin et al., 2021; Qiu et al., 2024). Most importantly, ESCs have the ability to fully develop into specific organs. In the study of *in vitro* culture of ESCs, Kasey Y C Lau et al.successfully obtained the beating heart tissue of mice in 5.5 ∼ 8.5 days of culture of two ectodermal lineages and post-implantation embryos of the pluripotency ESC lineage reconstructed by transcription factor-mediated induction. It is proved that ESCs have excellent plasticity, can develop on their own and produce complete embryo-like structures. (Lau et al., 2022). However, studies utilizing the property of direct differentiation of ESCs into cardiomyocytes for the treatment of cardiomyopathy are currently insufficient. Although ESCs, like iPSCs, have a strong ability to differentiate into cardiomyocytes, the role of paracrine activation of ESCs in cardiomyopathy therapy should likewise not be overlooked. A recent comparison of the roles of different stem cells in cardiomyopathy showed that ESCs, like MSCs, secrete extracellular vesicles (EVs) that are rich in proteins, metabolic intermediates, and nucleic acids, with mRNAs, microRNAs, and long-stranded noncoding RNAs predominating. ESCs reduce adverse cardiac remodeling after MI by downregulating fibrosis and increasing angiogenesis through paracrine effects (González-King et al., 2024).

### 2.4 Other stem cells

In addition to the above mentioned stem cells, vascular endothelial progenitor cells (EPCs) (Roura et al., 2007), monocytes, and brown adipose stem cells (BASCs) (Moon et al., 2022) have been mentioned in some of studies, we will not discuss cardiac stem cells (CSCs) here. EPCs exhibit functionally similar paracrine activity to MSCs and are mainly focused on hemotransfusion remodeling, e.g., EVs derived from EPCs are enriched in miR-126a-3p and a large number of vascular growth factors, including vascular endothelial growth factor (VEGF), stromal cell-derived factor-1 (SDF-1), C-X-C chemokine receptor type 4 (CXCR4), and endothelial nitric oxide synthase (eNOS), thereby promoting vascular growth (Yu et al., 2023). However, the instability of EPCs in therapy, the small research base, and the effectiveness of EPCs in clinical care that remains to be demonstrated limit the research and clinical translation of EPCs. Whether EPCs have the potential to be used in the treatment of cardiomyopathy is currently in doubt (Bianconi et al., 2018). Monocytes is an important type of white blood cells in the blood, belonging to the mononuclear phagocyte system. They mainly regulate inflammation activation and abatement, as well as the activation of the repair process of the damaged tissues. Although there are no separate studies on the use of monocytes for the treatment of cardiomyopathy, they still hold research value (Peet et al., 2020). BASCs have the potential to differentiate into CMs and are subject to mechanical stress like ESCs and iPSCs, e.g., in electrostatic spinning, BASCs can differentiate into myocardial-like structures and accelerate the growth of cardiomyopathy -injured blood vessels (Wei et al., 2023). A series of studies by Changyong Wang’s team have demonstrated that BASCs have excellent ability to regulate inflammation, anti-oxidative stress and improve cardiomyocyte apoptosis (Hao et al., 2017; 2018; Liu et al., 2023; Wang et al., 2014). Although the cell types mentioned above are therapeutically effective in the treatment of cardiomyopathy, the likelihood of achieving clinical translation remains low at this time due to the lack of current research.

## 3 Mechanisms and risks of stem cells therapy for cardiomyopathy

### 3.1 Stem cells differentiation and tissue regeneration

**FIGURE 1:**
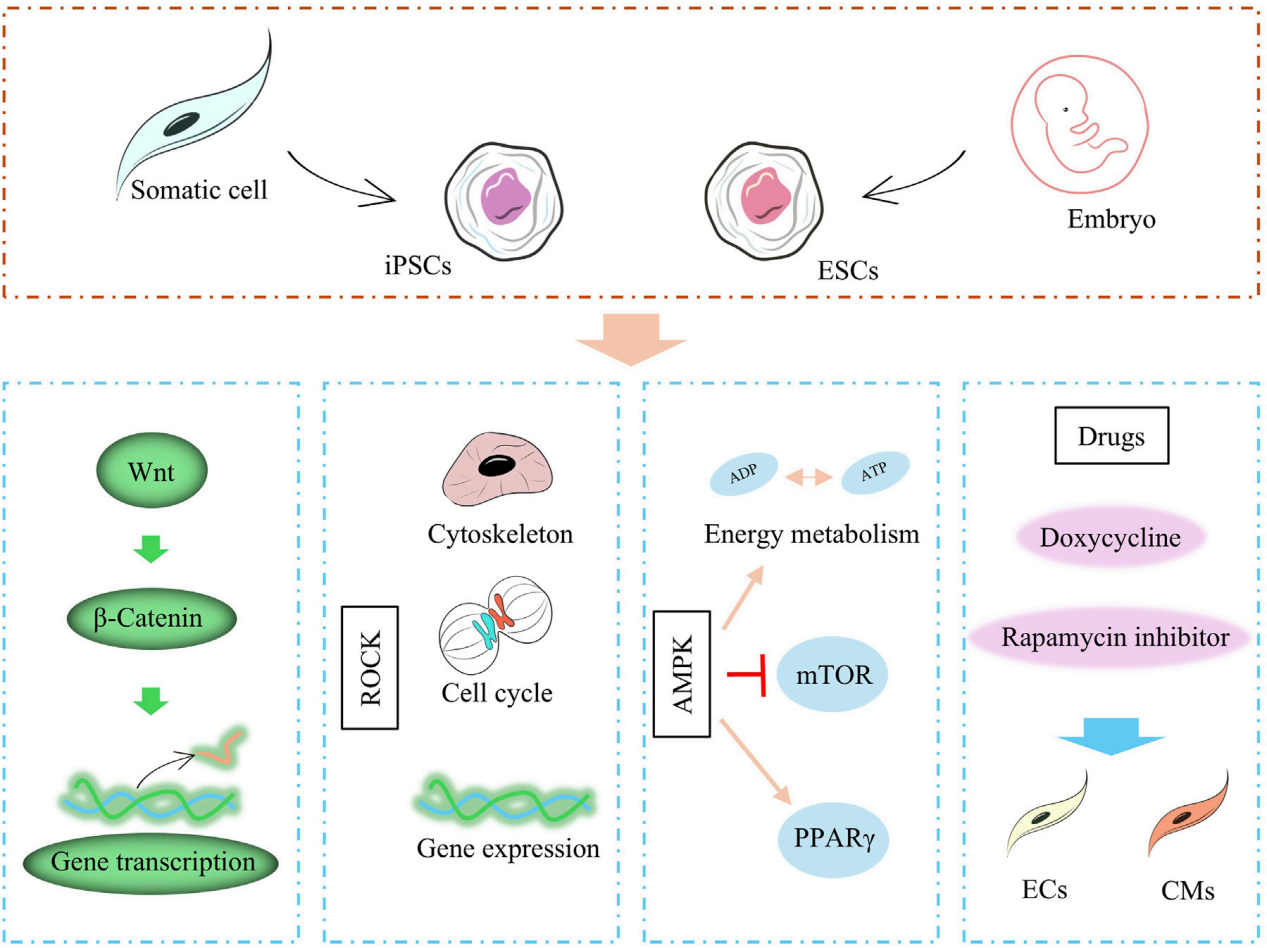
Factors affecting stem cell differentiation, including proteins, cytokines, related pathways, and drugs.

One of the important causes of death in patients associated with cardiomyopathy is CM loss and abnormal function caused by apoptosis of CMs during injury and during recovery, and usually accompanied by HF, and one of the key therapeutic ideas is to regenerate CMs to replace the damaged tissue. Since 2006, Takahashi and Yamanaka et al. have demonstrated by introducing four transcription factors, OCT3/4, c-MYC, SOX2, and KLF4, into mouse fibroblasts, they created mouse induced iPSCs and somatic cells that exhibit some properties similar to ESCs by expressing specific transcription factors and thereby (Takahashi and Yamanaka, 2006). Therefore, in this part, we mainly narrate the factors affecting the myocardial differentiation of ESCs and iPSCs. The developmental patterns of ESCs and iPSCs are extremely similar, and inducing the differentiation of ESCs and iPSCs to CM is an important step in this field of research. The CM-directed differentiation of ESCs and iPSCs is affected by growth factors, some of the signaling pathways as well as pharmacological factors (Figure 1).

Studies have shown that VEGF, bone morphogenetic protein 4 (BMP-4), activin A, and basic fibroblast growth factor (bFGF) are able to promote the differentiation of ESCs and iPSCs to CMs (Farboud et al., 2024). In addition, a number of signaling pathways, which are involved in multiple embryonic development, cell proliferation, differentiation, migration and maintenance of tissue homeostasis, greatly influence the differentiation process of ESCs and iPSCs. In the study by Loic Fort et al., CMs differentiation of iPSCs was influenced by Wnt signaling (cell proliferation, differentiation, migration related), and activation of Wnt signaling promoted mesodermal transformation of iPSCs (Fort et al., 2022). In Bin Jiang et al.'s study, CMs differentiation of iPSCs was affected by the activation level of Rho-associated protein kinase (ROCK) signaling (apoptosis-associated pathway), and inhibition of ROCK severely impaired the differentiation of iPSCs to CM (Jiang et al., 2022). AMP-activated protein kinase (AMPK) is an important energy, growth, and value-added regulatory pathway in cells. Studies have shown that the Thr172 site of AMPK is significantly phosphorylated during differentiation and that sustained activation of AMPK induces sirtuin-mediated deacetylation of histones and enhances the differentiation of iPSCs to the CM (Sarikhani et al., 2020).

Pharmacological factors also influence the differentiation process of ESCs and iPSCs and can induce the final course of stem cells differentiation through drug targeting. For example, the use of doxycycline to iPSCs can drive the transformation of iPSCs to endothelial cells (ECs) and stabilize the generation of induced ECs (iECs) (Luo et al., 2024). Inhibition of rapamycin signaling, on the other hand, could drive the maturation of iPSCs-CMs, which were prompted to exhibit contractile, metabolic, and electrophysiological properties similar to those of mature cardiomyocytes.

The study of ESCs and iPSCs in myocardial differentiation provides an important cell therapy strategy for the treatment of cardiomyopathy. The targeted differentiation of ESCs and iPSCs into CMs can be significantly promoted by growth factors, signaling pathways, and drug modulation. Factors such as VEGF and BMP-4, as well as the activation of signaling pathways such as Wnt, ROCK, and AMPK, play a key role in this process. Meanwhile, pharmacological factors such as doxycycline and rapamycin have also been shown to promote the transformation of stem cells to target cell types by modulating the differentiation process. Nonetheless, challenges in the clinical application of ESCs and iPSCs, such as differentiation efficiency, maturation of cells, and immune rejection issues, still need to be further optimized.

### 3.2 Paracrine effect

**FIGURE 2:**
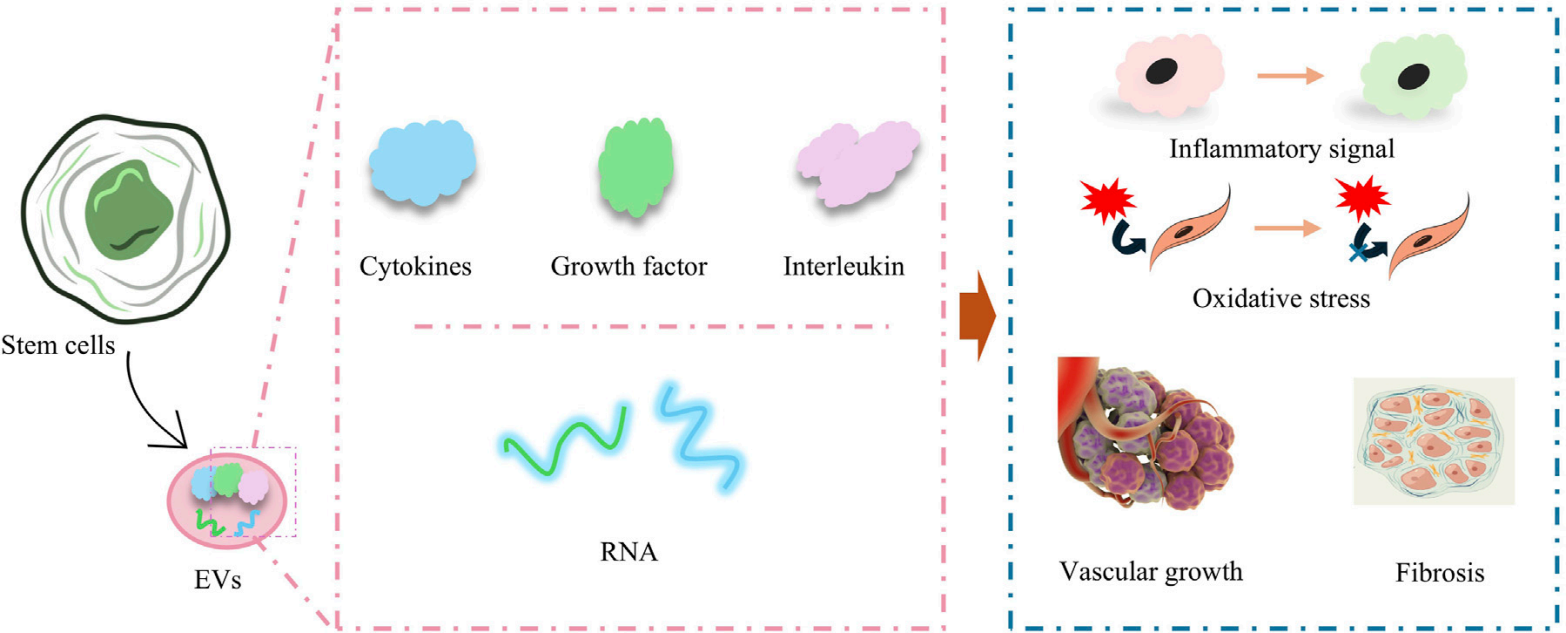
Paracrine effects of stem cells for amelioration of inflammation, mitigation of oxidative stress, promotion of vascular growth, and amelioration of myocardial fibrosis.

Paracrine effect is one of the most important core key links in the process of stem cells therapy for cardiomyopathy, in which the paracrine effect of MSCs as well as ESCs has been widely studied. A large number of studies have shown that the implantation of ESCs and MSCs in cardiomyopathy models induces the secretion of many EVs from ESCs and MSCs, which contain cytokines, growth factors, and various types of RNAs. EV influences the recovery of cardiomyopathy-injured tissues in multiple ways by regulating inflammatory signaling, oxidative stress, immunity, vascular growth, cellular value-added apoptosis and differentiation, and fibrosis (Figure 2) (Ren et al., 2024). In a recent study, umbilical cord mesenchymal stem cells (UCMSC)-derived small extracellular vesicles (sEVs) demonstrated potent anti-cardiac damage capacity in the treatment of radiotherapy-induced cardiac injury. The cardiac ejection function was protected by improving radiotherapy-injured cardiac energy metabolism, mitigating organelle structural damage in cardiac organs, alleviating oxidative stress state, and restoring cardiac calcium transients (Cao et al., 2024). Immune activation and suppression are important factors influencing the recovery of cardiomyopathy-injured tissues, and a recent study by Dashuai Zhu et al. demonstrated that direct cardiac injection of MSCs-derived EVs induced the activation of Forkhead box protein O3 (Foxo3) and promoted the expression of IL-10, IL33, and IL34 through protein phosphatase (PP)-2A/p-Akt/Foxo3 pathway, and greatly ameliorated the differentiation of Tregs from T cells. Interleukin 10, interleukin 33, and interleukin 34 (IL-10, IL33, and IL34), and induced the differentiation of Tregs from T cells, which greatly ameliorated the inflammation at the site of injury and promoted the repair of MI-injured myocardial tissue (Zhu et al., 2022).

A large number of RNAs contained in EVs also play a key role in cardiomyopathy recovery, such as microRNAs, miR-185, miR-221-3p, miR-19a, miR-144, miR-29c, miR-106a-363, miR-221, miR222, and miR-25-3p, by modulating the relevant gene expression and improved cardiac function after MI (Ala, 2023). Taking miR-106a-363 as an example, miR-106a-363 enrichment in EVs improved cell survival and contractility of elevated hypoxia-injured iPSCs-CM by upregulating cell cycle activators and cytoplasmic cleavage genes, while decreasing the level of myocardial fibrosis, and consequently, maintained the structure of the mouse left ventricle (LV), and increased the LVEF of the LV (Jung et al., 2021). Tzu-Lin Lee et al. also demonstrated that EVs secreted by adipose-derived MSCs contained a large amount of miR-221/222, which targeted and regulated the expression levels of PUMA or ETS-1 proteins through the miR-221/222/p38/NF-κB pathway and successfully reduced the apoptosis level and fibrosis level of CM (Lee T. L. et al., 2021).

MSCs and ESCs carry a variety of cytokines, RNAs, and growth factors through their secreted EVs, which can effectively regulate the processes of inflammatory response, oxidative stress, immune response, cell proliferation and apoptosis, thus accelerating the repair of myocardial injury. Due to the limitation of the isolation and purification technology of EVs, as well as the lack of understanding of the safety and efficacy of EVs, the conditions for clinical translation of EVs are still insufficient.

Although iPSCs, ESCs, and MSCs all have potent paracrine activity and exhibit beneficial effects on the recovery of injured cardiac tissue, the EVs secreted by different stem cells differ significantly. iPSCs/ESCs-EVs are enriched with a variety of functional signaling molecules as well as value-adding signals focused on influencing myocardial repair (Crow, 2019). For example, iPSCs/ESCs-EVs are enriched in transcription factors that maintain pluripotency such as Octamer-binding transcription factor 4 (OCT4), Sex-determining region Y-box 2, Nanog homeobox, etc., which may be delivered to the target cells through EVs and affect the gene expression reprogramming (Karagiannis et al., 2019; Lin et al., 2022). Some anti-apoptotic and pro-value-added signaling molecules, such as B-cell lymphoma-2, Insulin-like growth factor 1, and Fibroblast Growth Factor-2, promote cell survival and division (Karagiannis et al., 2019). Nucleic acid contents enriched in iPSCs/ESCs-EVs also influence the cellular value-added process, e.g., miR-302 family, miR-290/295 clusters, and targeted inhibition of differentiation-associated genes (e.g., p53, Rb) may induce cardiac progenitor cell proliferation (Ye e al., 2025). In contrast, MSCs-EVs focused on regulating the cardiac microenvironment and affecting the inflammatory response and vascular growth process in the heart. MSCs-EVs were enriched with a large number of anti-inflammatory and repair factors, with high expression of IL-10, Transforming Growth Factor - β (TGF-β), Human Growth Factor (HGF), VEGF, etc., which directly inhibit the Nuclear Factor-κB (NF-κB) inflammatory pathway and promote M2-type macrophage polarization (Hu et al., 2023; Yan et al., 2023). Some tissue repair-related proteins, such as Platelet-Derived Growth Factor (PDGF) and Insulin-like Growth Factor 1 (IGF-1), can synergistically promote neovascularization and extracellular matrix remodeling, and compared with iPSCs/ESCs-EVs, MSCs-EVs are almost do not contain transcription factors such as Oct4, and are dominated by mature tissue repair-related proteins (Guo et al., 2024; Xu et al., 2020). Meanwhile the function of nucleic acid contents contained within MSCs-EVs is focused on the protection of cardiac function, such as miR-1, miR-133 (promoting cardiomyocyte differentiation and electrophysiological stabilization), miR-21 (inhibiting fibrosis and targeting PTEN to reduce apoptosis), miR-126 (promoting angiogenesis), and the miR-29 family (targeting collagen genes COL1A1/COL3A1), reducing scar formation after myocardial infarction (Chen et al., 2017; Ding et al., 2024; Lee K. S. et al., 2021; Liu et al., 2020).

### 3.3 Risks of stem cells therapy for cardiomyopathy

The risks of stem cells therapy for cardiomyopathy are closely related to its type, source, preparation technique, and individual patient differences, with the core challenges being tumorigenicity, immune response, inconsistent efficacy, and unknown long-term safety.

Teratogenicity, the risk of tumor formation, is one of the central risks of stem cells use in cardiomyopathy treatment (Ko and Gelb, 2014). Among them, ESCs and iPSCs are prone to form tumors, including teratomas or other types of tumors, when ESCs and iPSCs are incompletely differentiated *in vivo* or proliferate abnormally *in vivo* due to their strong potential for multidirectional differentiation (Ash et al., 2017). While MSCs have a low risk of forming tumors (Gugjoo et al., 2019), prolonged *in vitro* culture or accumulation of mutations during induced differentiation also increases the risk of tumors in MSCs (Ash et al., 2017). Also, the effects of the stem cells transplantation process on other types of cells, including epithelial cells and vascular endothelial cells, increase the risk of tumorigenesis (Bartfeld et al., 2015; Mansour et al., 2018). For example, abnormal differentiation of stem cells leads to abnormal differentiation of epithelial cells, which affects the course of myocardial fibrosis (Tsukui et al., 2024). Stem cells have a strong role in promoting blood vessel growth, but abnormal blood vessel appreciation may lead to the development of hemangiomas. Rapid vascular appreciation also provides an ideal environment for tumorigenesis and further increases the risk of tumors treated with stem cells (Sun et al., 2023; Wei et al., 2021).

The immune response is a central issue that needs to be urgently addressed in stem cells therapy protocols and occurs primarily during allogeneic stem cells inhibition, with the consequences of the immune response being twofold (Ankrum et al., 2014). First, it has been shown that the immune response is one of the main reasons limiting stem cells therapy, including effects on stem cells activity, differentiation, and secretory functions (Burdick et al., 2016; Wang et al., 2024). For example, the release of inflammatory factors in the microenvironment after myocardial infarction severely affects the antioxidant, differentiation, and paracrine functions of Brown Adipose-Derived Stem Cells (BASC), which is beneficial to the graft survival and function of BASC by scavenging free radicals and ameliorating inflammation (Liu et al., 2023). Second, Cytokine storm from immune activation increases the risk of several complications in the transplanted subject, and prolonged immunosuppressive therapy increases the risk of host infection and tumorigenesis (Norelli et al., 2018). Although autologous stem cells theoretically have no or mild immune response, the difficulty in obtaining autologous stem cells, the tendency of stem cells to age, and the low efficiency of transplantation have limited the research and application of autologous stem cells (Golpanian et al., 2016).

## 4 Enhancing the effectiveness of stem cell therapy

Although stem cells have shown good therapeutic efficacy in the treatment of cardiomyopathy, they have long been limited by a number of factors and have not been able to realize the translation of therapeutic options into the clinic. First, the source of stem cells is difficult, and due to the ethical constraints of stem cells acquisition, ESCs can only be used in preclinical studies at present. Although iPSCs do not face ethical restrictions, current technology does not yet allow access to iPSCs that can be widely used, limiting the need for personalization in cardiomyopathy therapy. Second, various types of stem cells differentiate into CMs less efficiently and still have some functional differences from native CMs, such as contractility, secretory activity, and electrophysiological properties. Finally, injury during transplantation and post-transplantation immune rejection as well as an cardiomyopathy -injured environment can lead to poor survival, loss of function, and increased immune risk due to immune rejection after stem cells transplantation. The unpredictable unrestricted increase in stem cells after transplantation also increases the risk of tumorigenesis due to their potent value-adding and differentiation activity. Therefore, the use of stem cells for cardiomyopathy therapy still has many issues that need to be addressed. In order to address these issues, a large amount of research in recent years has focused on how to increase access to more efficiently differentiated and secreted stem cells, how to efficiently use stem cells in cardiomyopathy treatments and address the low survival rate of stem cells transplants, and how to reduce the immunological risks of stem cells transplantation. This includes the pre-treatment of stem cells, the use of biomaterials or nanotechnology to assist stem cells transplantation or the use of pharmacologically active molecules or proteins to assist in stem cells inhibition.

### 4.1 Construction and maturation of stem cells

#### 4.1.1 Pretreatment of stem cells

**FIGURE 3:**
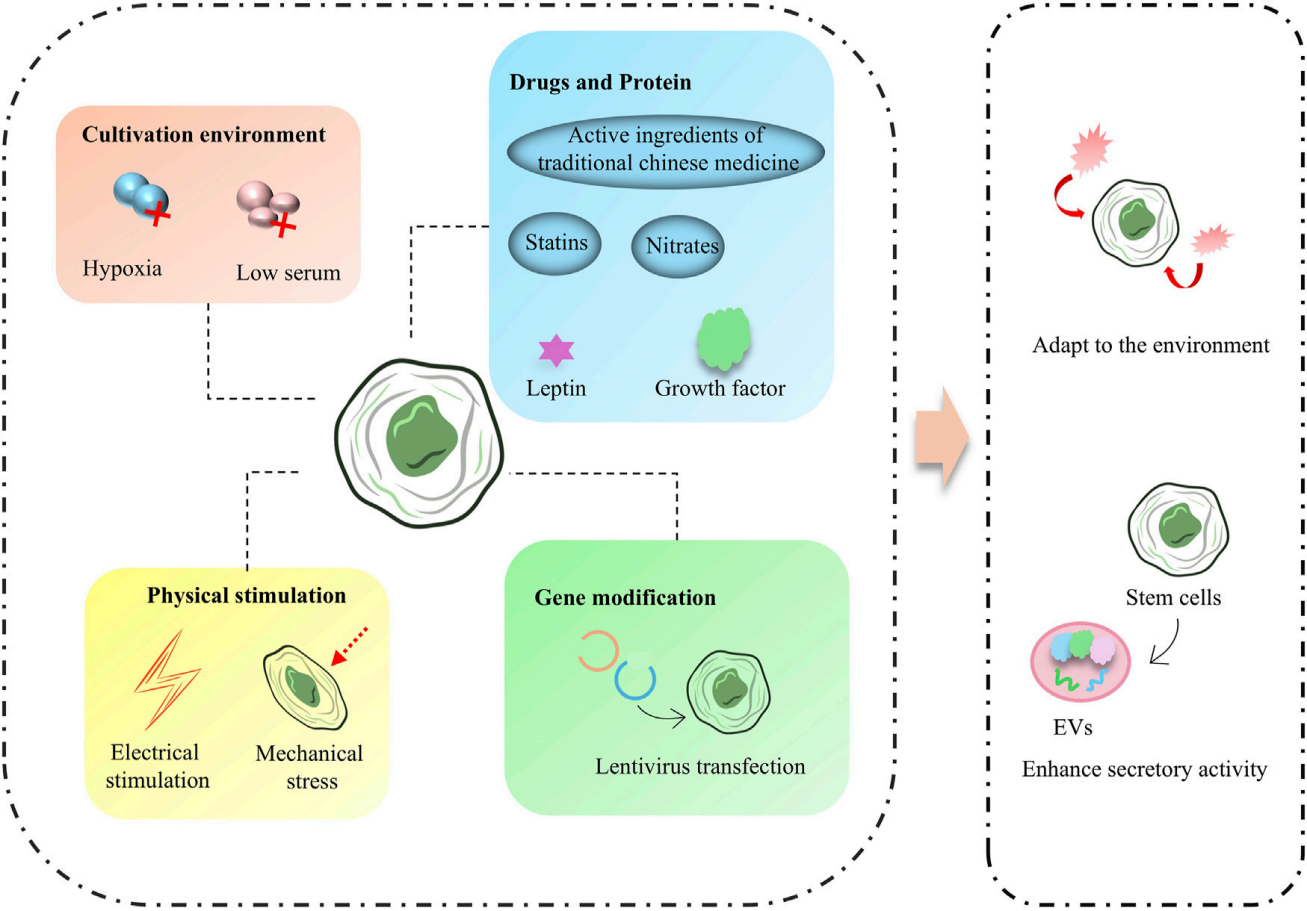
Stem cells pretreatment scheme and effect.

Pretreatment of stem cells is designed to improve their tolerance to graft injury, promote their differentiation, increase their activity, orconfer specific functions upon them, and treatments include hypoxia, serum deprivation, electrical stimulation, or treatment with biologically active drug/non-drug molecules. The ability of stem cells to combat injury and increase their activity can be increased by preconditioning, and this type of protocol is usually achieved by modulating stem cells sugar/oxygen metabolism-related genes and pathways, including some endogenous active molecules or metabolism-regulation-related drugs (Figure 3). Zheng Zhang et al. found that pretreatment of MSCs with an inhibitor of inositol hexakisphosphate kinases significantly increased MSCs’ resistance to hypoxic and ischemic environments and increased MSCs’ survival after transplantation into MI tissues (Zhang et al., 2014). Some molecules that improve mitochondrial function also enhance the survival of MSCs, such as leptin-treated MSCs that maintain mitochondrial integrity and cope with various stresses (Yang et al., 2018). Growth differentiation factor 15 increases the tolerance of MSCs to hypoxic environments by improving mitochondrial function and significantly increases the survival of MSCs after cardiac transplantation (Huang et al., 2024). To counteract ROS in the cardiomyopathy-injured environment, Chan Wu et al. developed a bradykinin peptide to pretreat MSCs and successfully reduced the expression of caspase 3 and Bcl-2-associated X protein (Bax) and inhibited apoptosis in MSCs in the ROS environment (Wu et al., 2021).

Some treatment protocols can confer specific secretory activity to stem cells. Hypoxic-ischemic preconditioning is one of the widely used preconditioning protocols for stem cells (Zhao et al., 2022). Under the hypoxic-ischemic environment, the tissue repair-related activity of stem cells is activated in advance, exerting a strong pro-neoplastic capacity. This is conducive to the reduction of MI infarct area and acceleration of neovascularization (Xiong et al., 2022a). Some drugs widely used in cardiovascular diseases are also used in stem cells preconditioning. For example, EVs secreted by bone marrow MSCs treated with atorvastatin, a clinical drug that improves blood lipids, regulate macrophage phenotype through the miR-139-3p/Stat1 pathway and thus have excellent therapeutic and repair effects in acute myocardial infarction (AMI) (Ning et al., 2023). Meanwhile, long chain non-coding RNAs contained in EVs secreted by MSCs pretreated with atorvastatin. e.g., miR-675 also improved the prognosis of AMI treatment by promoting vascular growth (Huang et al., 2020). Some analogous nitrate drugs are also beneficial in enhancing the secretory activity of stem cells, and in a recent study by Zhao-Ting Gong et al., Nicorandil-pretreated MSCs improved macrophage phenotype and enhanced myocardial repair after MI by modulating miR-125a-5p/TRAF6/IRF5 signaling (Gong et al., 2024). Several physical protocols are also beneficial in promoting the fractionation of stem cells to CMs and conferring cardiomyocyte-like secretion and functionality. iPSCs pretreated with electrical stimulation exhibited a more CM-like secretory capacity through activation of Ca^2+^/PKC/ERK (Ma et al., 2018). Stem cells differentiation also induces the effects of mechanical stress in the culture environment, and one study showed that periodontal ligament-derived stem cells (PDLSC) pretreated with mechanical tensile stress were able to produce more ganglionic actin and secrete nitric oxide (Pelaez et al., 2017).

In addition, some pretreatment methods based on the active ingredients of traditional Chinese medicine/herbal medicine have shown promising results in the development of stem cells (Huang et al., 2018). Pretreatment regimens in this category usually improve the ability of stem cells to cope with stressful conditions through oxygen cycle-related pathways. For example, MSCs pretreated with the traditional Chinese medicine Tongxinluo (TXL) showed excellent secretory activity in secreting anti-apoptotic and anti-inflammatory molecules (Xiong et al., 2022b). MSCs treated with tachyphylaxis also exhibited longer cardiac mainstreaming capacity, while enhanced paracrine activity promoted cardiomyocyte value-addition (Ruan et al., 2018). Artemisinin, an antimalarial drug from traditional Chinese medicine, reduced the ROS level and decreased the expression of Caspase-3 in MSCs during pretreatment of bone marrow MSCs, effectively improving the apoptosis level of MSCs in a ROS environment (Fang et al., 2019).

#### 4.1.2 Genetic modification

Genetic modification of stem cells also aims to improve cell survival and ensure stem cells activity after transplantation and also provides theoretical and practical ideas for human intervention in stem cells differentiation. In order to combat apoptosis after stem cells transplantation, researchers usually choose to gene-edit stem cells to make them express a large number of proteins, such as apoptotic proteins, including growth factors, interleukins, and some anti-cellular senescence factors (Raziyeva et al., 2020), or overexpression of some miRNAs to play the role of inflammation inhibition and repair. Yun Zhao et al. successfully obtained MSCs with high expression of growth differentiation factor 11 (GDF11) by genetic lentiviral transfection, and explored the therapeutic efficacy of GDF11 to enhance the therapeutic efficacy of MSCs through the TGF-β receptor/Smad2/3/YME1L-OPA1 pathway (Zhao et al., 2020). The implantation of some similar growth factors such as HGF (Zhao et al., 2016), IGF-1 overexpression genes has also conferred a similar high survival rate of stem cells transplantation (Lin et al., 2020). Some studies have also focused on combating inflammation, mainly interleukins, including IL-7 (Haneef et al., 2018), IL-10 (Meng et al., 2018) and IL-33 (Chen et al., 2019), and some miRNAs that inhibit inflammation. High expression of anti-inflammatory interleukins enhanced the therapeutic capacity of MSCs after transplantation, mainly in terms of improved inflammation after cardiac injury, including modulation of inflammatory factor expression and of macrophage phenotypes, and consequently significantly reduced infarct size (Chen et al., 2019). Some inflammation-suppressing miRNAs showed similar therapeutic effects, e.g., miR-15a/b, miR-19a/b. miR-15a/b could enhance cardiac repair in MI mice via VEGFR-2/PI3K/Akt pathway (Chen et al., 2021; Tu et al., 2019).

Stem cells pretreatment, as a strategy to enhance the effectiveness of stem cells transplantation, is of great significance in improving the therapeutic efficiency of stem cells. Through different treatments, it can effectively improve the tolerance of stem cells to environmental damage, enhance their survival rate, promote differentiation, and even endow them with specific secretory activities. Treatments such as hypoxia, serum deprivation, electrical stimulation, and pharmacologic preconditioning can significantly improve stem cells function. Hypoxic-ischemic pretreatment activates the repair potential of stem cells in advance, which helps accelerate neovascularization and myocardial repair, while drugs such as atorvastatin improve repair after myocardial infarction by modulating stem cells secretions. Some traditional Chinese medicines and their active ingredients have also shown promising results in stem cells preconditioning studies and can enhance the therapeutic potential of stem cells by improving oxygen circulation and stress response. Although these preconditioning regimens still face some challenges in clinical application, they undoubtedly provide new ideas and methods for stem cells therapy of cardiac diseases.

### 4.2 Stem cells transplantation optimization

**FIGURE 4:**
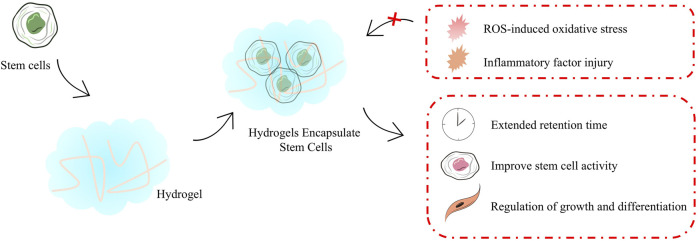
Emerging delivery strategies for stem cells.

Stem cells transplantation optimizes the process of transplantating cells that require protection to safeguard them from mechanical damage during transplantation. As the field of biomaterials continues to evolve, a number of stem cells transplantation strategies with stem cells protection have been progressively developed, including hydrogels (Figure 4). These transplantation protocols aim to enhanced the therapeutic effect of stem cells in cardiomyopathy. Hydrogels are among the most common biomaterials used in stem cells transplantation for cardiomyopathy therapy and provide important protocol support to advance the clinical translation of stem cells therapy, mainly playing an important role in providing a supportive environment (Table 1) (Roshanbinfar et al., 2023), regulating stem cells growth and differentiation, mimicking the ECM environment, combining drugs for synergistic therapies, reducing immune rejection, and restoring electrophysiological properties. Hydrogels for stem cells delivery typically have excellent injectable properties, *in situ* gelation effects, and good biocompatibility. *In situ* gelation can be achieved by a variety of factors, including temperature, time, and proteins in the organism (Gao et al., 2020). For example, hydrogel patches constructed by modulating the formation time of hydrogels and incorporating the natural structure of the pericardial cavity successfully reduced the immune response and increased the retention time of stem cells in the pericardial cavity during the transplantation of iPSCs or MSCs (Zhu et al., 2021). Temperature is likewise an available condition for hydrogel formation, as in the case of hydrogels constructed on the basis of chitosan/dextran/β-glycerophosphate, which formed hydrogels and prolonged myocardial retention time and facilitated myocardial differentiation of umbilical cord MSCs at post-injection body temperature temperatures (Ke et al., 2020). Utilizing good degradability also provides a safety basis for *in vivo* implantation of hydrogels, which is usually achieved using biologically responsive breakable cross-linking, e.g., 2-ethyl-2-oxazoline and 2-butenyl-2-oxazoline cross-linking of hydrogels via dicysteine carrying MSCs significantly increased pro-vascular genes in MSCs after implantation expression and degraded and metabolized out of the body after transplantation (You et al., 2021). Regulation of stem cells growth and differentiation is also one of the important functions of hydrogels, and many previous studies have shown that encapsulating stem cells in hydrogels facilitates cardiac differentiation of stem cells, including adipose-derived MSCs (Wang et al., 2014) as well as iPSCs (Kerscher et al., 2016). Taking polyethylene glycol-fibrin hydrogel as an example, the well-biomimetic 3D structure provides environmental support for the development of iPSCs to have cardiac tissue structure and function (Kerscher et al., 2016).

**TABLE 1:** Types and roles of hydrogels for stem cells protection.

Hydrogel function	Cell types	Hydrogel material	Diseases	References
Supportive environment, eliminate ROS	MSCs	HA	HF	Yue et al. (2024)
Simulation of ECM environment with loaded functional nanoparticles	MSCs	Natural Alginate Hydrogel	MI	Liu et al. (2023)
Enhancement of stem cells secretory activity	iPSCs	HA	MI	Zhu et al. (2021)
Regulates stem cells growth and differentiation, loads therapeutic drugs	hPSC	Collagen Hydrogel	MI	Goldfracht et al. (2020)
3D Printing, Protecting Stem Cells	iPSCs	3D printed bio-inks	MI	Noor et al. (2019)
Light curing, delivery of EVs	iCM-EVs	Light curing	Inhibition of myocardial adhesion	Wang et al. (2023)
Conductive biomaterials, protection of stem cells	hPSC	Polypyrrole chitosan	MI	Yan et al. (2024)
Improvement of electrical signals, delivery of stem cells	iPSCs	Poly (3,4-ethylenedioxybiphenyl) polystyrene sulfonate	MI	Roshanbinfar et al. (2024)

In addition, the diversity of sources for constructing hydrogel substrates provides a material basis for the construction of hydrogel functionality and the possibility of modulating the ECM, thereby increasing the therapeutic efficacy of stem cells. The development of cardiomyopathy is usually accompanied by inflammatory activation of the ECM and overloading of ROS, which greatly affects the engraftment and activity of stem cells. Therefore, a great deal of research has focused on how to utilize hydrogels to load stem cells while empowering the hydrogels to improve the ECM. To achieve this function, Wang C et al. loaded fullerol nanoparticles in alginate hydrogels and effectively scavenged superoxide anion and hydroxyl radicals in ECM and enhanced the therapeutic capacity of brown adipose stem cells (Hao et al., 2017). Subsequently, the team optimized the protocol several times over the years, and in a recent study, the team successfully encapsulated bifunctional Au@Pt core-shell nanoparticles into a hydrogel, which endowed it with free radical scavenging ability and also opened up the possibility of improving the electrophysiological properties of the heart by the hydrogel. In the MI model of mice, the composite hydrogel loaded with brown adipose stem cells successfully enhanced the angiogenesis and improved electrical signal conduction in the heart by brown adipose stem cells. In a mouse MI model, the composite hydrogel loaded with brown adipose stem cells successfully enhanced the pro-angiogenic ability of brown adipose stem cells and improved cardiac electrical signaling (Liu et al., 2023).

The development of injectable hydrogels has provided some viable options for mimicking ECM *in vitro*, but decellularized extracellular matrix (dECM), obtained by directly removing cells, appears to provide more favorable conditions for stem cells engraftment. In 2022, Jonathan H Tsui et al. developed a porcine heart-derived dECM hydrogel and combined it with reduced graphene oxide to form a hybridized hydrogel that was able to maintain cardiac electrophysiological properties while also significantly enhancing contractility and increasing expression of genes related to the regulation of contraction in iPSCs-CMs (Tsui et al., 2021). It was also found that the preparation of porcine heart-derived dECM into granular hydrogel-embedded iPSCs was able to maintain the optimal viability of iPSCs, which may be a function of the fibronectin enriched in dECM (Shaik et al., 2024). Moreover, dECM, which is rich in various cytokines and growth factors, is more effective in improving fibrosis and promoting capillary growth compared to other hydrogels (Shaik et al., 2023). Although dECM shows great potential in maintaining stem cells function, the acquisition of hydrogels in this manner is currently in the laboratory research stage and subsequent dissemination may be limited by the process of dECM acquisition and preparation as well as ethical constraints.

The loss of CMs following myocardial injury and induced consequent fibrosis leads to abnormal cardiac electrical signaling, therefore, abnormal cardiac electrical signaling function should not be ignored while replenishing CMs. In many recent studies, researchers have endowed hydrogels with excellent conductive properties and play an important role in maintaining normal cardiac rhythms. The conductive properties of hydrogels can usually be achieved by the presence of organic polymers with active electrons or metal ions. For example, the incorporation of Au@Pt core-shell nanoparticles in hydrogels, as mentioned earlier, has conferred excellent electrical signaling capabilities to hydrogels (Liu et al., 2023). Or other forms of gold nanoparticles, such as positively charged gold nanoparticles carrying gold chloride reduced by branched polyethyleneimine (Roshanbinfar et al., 2023). In a recent report, Kaveh Roshanbinfar et al. developed an injectable collagen-PEDOT: PSS (poly (3,4-ethylenedioxythiophene) polystyrene sulfonate) hydrogel was developed that protects infarcted hearts from ventricular tachycardia (VT) and binds to hiPSCs-cardiomyocytes to promote partial cardiac remuscularization (Roshanbinfar et al., 2024).

A number of different morphological matrices may play unexpected roles in stem cells transplantation. Xu et al. significantly increased the activity of iPSCs and efficiently differentiated them into cardiac tissues by loading them in hyaluronic acid hydrogel microspheres to mimic the natural process of early embryonic development (Xu et al., 2021). Protein nanosheets developed by Elijah Mojares et al. enabled stem cells to maintain the stem cells phenotype and exhibit potent secretory activity in long-term culture due to their unique interfacial mechanical properties (shear modulus >10–30 mN m).

With the development of biomaterials technology, hydrogels have shown great potential as important carriers in stem cells transplantation. Hydrogels not only provide physical support and mimic the ECM environment but also provide an ideal microenvironment for stem cells therapy. They regulate the growth and differentiation of stem cells as well as promoting the recovery of cardiac electrophysiological properties, and have gradually become an important part of cardiomyopathy protocols for stem cells therapy.

## 5 Conclusion

Cardiomyopathy is a serious threat to human health, and traditional treatments have obvious limitations in repairing damaged myocardium and restoring cardiac function. The emergence of stem cells therapy has brought new hope for cardiomyopathy. In the course of in-depth research on various types of stem cells therapy for cardiomyopathy, we have gained important results and profound understanding in various aspects. Induced iPSCs, MSCs, ESCs and other stem cells have their own unique advantages and potentials in the treatment of cardiomyopathy. The strong differentiation ability of iPSCs, which come from abundant sources and do not have the worry of immune rejection, provides strong support for cardiomyocyte regeneration. Although MSCs cannot be directly differentiated into cardiomyocytes, they can improve the myocardial microenvironment and reduce inflammation and fibrosis by virtue of their significant paracrine effects. The high proliferation and differentiation potential of ESCs makes them occupy an important position in the field of myocardial repair, and their paracrine effects should not be underestimated. These stem cells types provide possible pathways for improvement and repair of cardiac function from different perspectives. At the level of therapeutic mechanism, in terms of myocardial repair and regeneration, research on the regulatory mechanism of ESCs and iPSCs differentiation into cardiomyocytes has made progress, and the clarification of growth factors, signaling pathways and drug effects has provided a theoretical basis for directional induction of differentiation. In terms of paracrine effect, the rich content of EVs secreted by MSCs and ESCs has been gradually revealed to regulate the repair of myocardial injury, which fully demonstrates the key significance of intercellular interaction in cardiac repair. In view of the many problems faced by stem cells therapy, the exploration of new technologies has brought light to its clinical transformation. Various means of stem cells pretreatment and genetic modification can effectively enhance the tolerance, activity and function of stem cells, and significantly increase their application value in cardiomyopathy therapy. In terms of transplantation optimization, the development and application of biomaterials such as hydrogels have played an important role in protecting stem cells, promoting their action and improving cardiac electrophysiological properties by constructing a suitable microenvironment for cell transplantation, which provides a new direction for future research despite the limitations of dECM. However, stem cells therapy for cardiomyopathy is still in the developmental stage, and there is still a long way to go before widespread clinical application. Future research should focus on further improving the differentiation efficiency and functional maturity of stem cells, in-depth investigation of their long-term safety and efficacy *in vivo*, optimization of transplantation technology and biomaterials, strengthening of multidisciplinary cross-cooperation, integration of basic research and clinical practice, and promotion of stem cells therapy from the laboratory to the clinic, which will bring practical, safe and efficient new therapeutic options for cardiomyopathy patients, and is expected to reshape the landscape of cardiomyopathy treatment and improve patients’ prognosis and quality of life. It is expected to reshape the treatment pattern of cardiomyopathy, improve patients’ prognosis and quality of life, and open a new chapter in the treatment of cardiovascular diseases.
